# A Nature’s Way—Our Way Pilot Project Case Assemblage: (Re)Storying Child/Physical Literacy/Land Relationships for Indigenous Preschool-Aged Children’s Wholistic Wellness

**DOI:** 10.3390/children10030497

**Published:** 2023-03-02

**Authors:** Kathryn Riley, Amanda Froehlich Chow, Kathleen Wahpepah, Natalie Houser, Mariana Brussoni, Erica Stevenson, Marta C. Erlandson, M. Louise Humbert

**Affiliations:** 1 School of Public Health, University of Saskatchewan, Saskatoon, SK S7N 2Z4, Canada; 2 Saskatoon Public Schools, Saskatoon, SK S7K 1M7, Canada; 3 College of Rehabilitation Sciences, University of Manitoba, Winnipeg, MB R3E 0T6, Canada; 4 Department of Pediatrics and the School of Population and Public Health, University of British Columbia, Vancouver, BC V6T 1Z3, Canada; 5 Saskatoon Tribal Council, Saskatoon, SK S7N 4S1, Canada; 6 College of Kinesiology, University of Saskatchewan, Saskatoon, SK S7N 5B2, Canada

**Keywords:** relational ontologies, new materialism, Indigenous Knowledges, storytelling, movement pedagogies

## Abstract

Physical literacy (PL) is gaining more attention from educational policy-makers, practitioners, and researchers as a way to improve health and wellness outcomes for children and youth. While the development of PL is important for early years children, there is limited attention in the literature that explores the political, cultural, and social discourses imbued in colonialism that implicate how PL is actualized in Indigenous early childhood education (ECE) contexts. This case assemblage explores how the culturally rooted, interdisciplinary, and community-based PL initiative, Nature’s Way–Our Way (NWOW), negotiated movement with three early childhood educators in the pilot project with an early childhood education centre (ECEC) in Saskatchewan, Canada. Through postqualitative approaches to research, this case assemblage adopts new materialist methodologies to show how the natural order of knowing in movement was disrupted through moments of rupture generating stories of PL to encompass radical relationality with land. As land becomes a vital and lively part of PL storying, it can function as an important protective factor for Indigenous preschool-aged children’s wholistic wellness.

## 1. Introducing Possibilities for Different Stories of Physical Literacy

What is already known on this topic:

1. Developing ph physical literacy (PL) from a young age promotes physical activity (PA), health, and wellness across the lifecourse [1,2,3].
2. Developing A strong sense of cultural identity and connection to family, community, spirituality, and land are protective factors for Indigenous social and emotional wellness [4].

What this article adds:

1. Dwelling with the tensions of political, cultural, and social discourses that impact how PL is actualized.
2. PL rooted in cultural identity and connection to family, community, spirituality, and land is a protective factor for Indigenous wholistic wellness.

*“Relationships do not merely shape reality; they are reality”*.

[5] (p. 7)

The International Physical Literacy Association (IPLA) defines physical literacy (PL) as the “motivation, confidence, physical competence, knowledge, and understanding to value and take responsibility for engagement in physical activities for life” [6]. Enabling individuals to make healthy and active choices through the motivation and capacity to understand, communicate, apply, and analyze different forms of movement across a variety of environments and sociocultural contexts, PL is a concept grounded in the wholistic development of the individual through a focus on four interconnected domains (affective, physical, cognitive, and behavioral) [7,8,9,10,11,12]. PL, therefore, is the gateway to physical activity (PA) throughout the lifecourse [13,14]. Further, as dominant discourses define what being physically active means while implicating continued engagement in PA, PL has the capacity to respond to the status quo of normalizing educational processes that mobilize and make relevant some storylines while eclipsing others [15,16,17,18,19,20,21,22]. PL does this through its embodied dimension, tapping into the full range of individual potential regardless of ability [10]). Thus, helping individuals become richer persons in themselves regarding what they know of the world, PL has a significant impact on self-image, self-esteem, and the development of a positive sense of self [11,12]. 

What happens, however, when PL meets the universalizing effects of colonialism? Considering Margaret Whitehead’s [10] important provocation querying whether PL is a culturally grounded or universal concept, in this article, we ground PL as contextualized, emplaced, and situated within Indigenous relationships with land. In doing so, we seek to respond to universal and colonial logics that commodifies, objectifies, and developmentalizes children’s movement [23,24,25] while simultaneously severing the child away from the natural world [26,27,28,29]. For example, the ‘obsession with data’, articulated as ‘testing’, is a set of calculative tactics in education that prioritise objectives, outcomes, standards, high-stakes testing, competition, achievement, and performance within liberal, capitalist notions of community, often without collective responsibility. To trouble orthodox ways of understanding movement phenomena and offer a way forward for thinking differently about universal and colonial logics of movement, we open possibilities for PL in this article through complex thinking that embraces affective, aesthetic, sensory, non-rational, and non-linear ways of moving [30,31]. We do this through the (re)configuration of the individual (educator and child) as emergent within radical relationality of highly interconnected and interdependent social–cultural–material environments [32,33]. Resisting reductionist forms of movement that seeks certainty and generalizability, we depart from a focus on understanding the individual as something that ‘is’ through essence to focus on how the individual comes into being through notions of becoming [34].

The radical relationality that we take up in this article is conceptualized through Karen Barad’s [32] ethico-onto-epistemology. Proposing a different approach to empiricism (about knowledge) that departs from Cartesian knowledge claims that separate the nature of reality (ontology) and our knowledge of it (epistemology), Barad’s ethico-onto-epistemology is defined by connectivity rather than hierarchy and separation. For Barad, we do not make sense of the world through knowledge acquisition (e.g., Cartesian representational knowing) but through the senses sensing; we are of the world, emerging as part of the world through discursive (social) processes and materiality (nature), forming moment-to-moment worldmaking practices. Barad argued that matter and culture are not discrete entities nor in opposition to one another, but rather, they are mutually constitutive. Thus, the ‘ethico’ in ethico-onto-epistemology means that relationships do not exist because we are accountable to them through specific actions (as behavioural change models might purport within the Canadian Physical Activity Guidelines for the Early Years by the Canadian Society for Exercise Physiology [2021] and ParticipACTION Early Years Guidelines [2016]) [35] but that worldings are constituted by these relationships [32,36]. Understanding that we are not separate and discrete from the units of inquiry in this article, we do not stand at a distance to report our findings through a linear process of ‘methods’ producing ‘data’ or ‘results’ [37,38,39]. Rather, mapping the conditions of entanglement within postqualitative methodological response-abilities, we take up a renewed vocabulary and conceptual toolbox to bring into question the taken for granted assumptions in normalizing movement logics. Thus, our methodological agenda in this article does not follow conventional structures or framings through a hierarchical logic of representation (e.g., interpretation and analysis as conventionally understood). As we resonate and relate with the educator stories (data), we enact a thinking-with the research participants, the land-based context in Saskatchewan, Canada, and various literatures to generate different lines of flight and thus, different stories, assemblages, and worldings of PL theorizing. Moreover, we resist the (fore)closure of possibilities for PL through a conventional ‘conclusion’ in this article; but in offering an ‘in-conclusion’ that maps the implications of PL understood through complexity thinking’s radical relationality, we move towards open-ended possibilities that may reside in (and beyond) what has been discussed and actualized in this article [40].

This methodological (re)framing of PL through complexity thinking’s radical relationality is put to work in this article through a focus on the culturally rooted, interdisciplinary, and community-based PL initiative, Nature’s Way-Our Way’s (NWOW) pilot project. Adopting new materialist approaches to understanding the world [32,33,41,42], we show how a child/PL/land assemblage emerged through the stories of three early childhood educators participating in the pilot project. We acknowledge Indigenous scholarship has a long history of agent ontologies and ethics of earthly materiality, well before Eurocentric/continental philosophy of new materialism [43,44,45,46,47]. Yet new materialism provides a useful and important methodology to explore relational ontologies from settler positionalities in avoiding the appropriation of Indigenous Knowledges. As new materialism is set within relational ontologies that suggest there is no separation between the human (participant) and what is studied (object), we depart from the idea of a ‘case’ as an object of inquiry that can be represented or explained in this article. Rather, we offer a case assemblage that is relationally inclusive of all bodies (human, animal, and earthly materialities of the natural world) and discursive practices (political, sociocultural, and ethical constructions) [48,49]. Concentrating on assemblages of human and nonhuman bodies together producing the world is an effort to generate different ways of storying PL, ways that are relevant and meaningful for Indigenous preschool-aged children within colonial logics imbued within dominant political, cultural, and social discourses. The aim of this article, therefore, is to show how pedagogical events in the pilot project produced ‘moments of rupture:’ the small fissures in orthodox ways of understanding movement phenomena that initiate a minor, dissident flow that spreads away from spaces housing dominant discourses [50,51]; for instance, universal and colonial logics of movement that separates the moving child from the land upon which they move. To provide a visual of a moment of rupture—a break in the natural order—consider a broken branch that has fallen from a tree lining a riverbank. As this branch crashes down and displaces the sand and water, it will cause the river to flow in different ways within the larger body of water. In NWOW’s pilot project, moments of rupture provide different ways of storying PL within broader political, cultural, and social discourses. Showing how land becomes a vital and lively part of PL storying in this article, our efforts seek to illuminate Indigenous cosmologies (e.g., human/land relational reciprocity) in naming particular realities and corresponding ideologies missing in mainstream models of Western education to support Indigenous self-determination and sovereignty for wholistic health and wellness across the lifespan [52,53,54,55,56]. 

## 2. The Background of Nature’s Way–Our Way

Created by a team of First Nations, Métis, and Settler researchers and community members, NWOW is implemented in nine Indigenous and non-Indigenous early childhood education centres (ECECs) across Saskatchewan, Canada. The initiative emerged in 2021 from the Healthy Start/Départ Santé project that was developed in 2012 to improve healthy eating, PL, and PA in Anglophone and Francophone ECECs in Saskatchewan and New Brunswick, Canada [57]. To improve the knowledge, attitudes, and self-efficacy of early childhood educators, directors, and families towards the development of the whole child, Healthy Start/Départ Santé sought to influence factors across socio-cultural-material-political domains, namely intrapersonal (e.g., eating and PA behaviours of children), interpersonal (e.g., educators and parents), organizational (e.g., ECEC), community (e.g., community organization involvement), physical environment (e.g., built and natural), and political levels (e.g., policies). Implemented in 140 licensed childcare centres, the results of Healthy Start/Départ Santé indicated increased PA and healthier eating among children who had participated in the study [57,58]. However, through Healthy Start/Départ Santé’s focus on Western practices and resources, there was a noticeable lack of Indigenous Knowledges within the project that led to the development of NWOW. Within NWOW, a series of PL-enriched movement resources were co-created amongst the team of First Nation, Métis, and Settler researcher and community members. Elder Kathy created the first draft of the PL-enriched movement resources representing traditional teachings (storytelling) and games, which were then creatively depicted on the resources by an Indigenous artist. PL from Western perspectives was then incorporated to weave Indigenous and Western Knowledges together in the development of PL for preschool-aged children through Indigenous games, activities, cultural connections, and traditional teachings [59].

Recognizing that the early years is a crucial time to develop PL [1,2,3], NWOW is positioned to influence physical behaviors and psychological and social factors through PL-enriched movement opportunities for preschool-aged children that promote resilience, creativity, and joy in movement while also providing opportunities for preschool-aged children to meet daily recommendations of PA as an important protective factor for health and wellness. Moreover, NWOW recognizes the sacred relationship many Indigenous peoples have with the land and how this connects to their wholistic wellness, paying attention to how human/land relationships were reorganized through colonialism removing Indigenous peoples from the land and stabilized through continuing colonial structures [36]. While Indigenous communities across Saskatchewan, Canada and throughout the world have demonstrated unwavering resilience that has led to adaptations, persistence, and transformational responses to colonialism, Indigenous peoples’ experiences of poor health outcomes cannot be separated from ongoing structures of colonialism [60,61]. As a praxis of resistance to settler colonialism, NWOW supports early childhood educators, families, and preschool-aged children to actualize PL through a focus on land relations in how children move in the ECEC and at home while also seeking to influence policies within the ECEC community [62,63].

As we discuss in this article, NWOW’s pilot project commenced with an ECEC in an original and mixed income neighborhood of Saskatoon in the spring of 2022 and involved a ten-month collaboration with three early childhood educators and seven Indigenous preschool-aged children and their families. The first phase of the collaboration involved professional development pertaining to PL philosophy, theory, practice, and how PL-enriched movement opportunities can be implemented through Indigenous games, activities, cultural connections, and traditional teachings, as depicted in a range of NWOW resources. For the full range of NWOW’s PL-enriched movement resources, please see: https://tinyurl.com/896r56p6. As part of the broader goals of improving children’s PL through repeated implementation and careful scaffolding of teachers’ and learners’ encounters with the resources, the pilot project focused on educator self-efficacy to role model and promote PL to then promote the PL of the children in their care. 

## 3. A New Materialist Mapping of Methods in NWOW’s Pilot Project 

In this article, we move beyond a purely discursive gaze focused upon social dynamics to draw on the concrete, complex materiality of bodies immersed in social relations of power [32,33,41,42]. While we will always be influenced by the social worlds in which we inhabit through materialist accounts, we are focusing on the interconnected relationship between matter inside and outside of bodies through the preconscious capacity for the body to act and be acted upon [64]. In other words, as bodies are affected by stimuli in the environment, bodies simultaneously affect the environment. Thus, from a new materialist perspective, knowledge acquisition and meaning making is not produced through a detached and separate human (participant) learning about the world (object), but rather, worldmaking (knowing/being/thinking/doing/feeling) occurs through the products and producing of environments as human and nonhuman bodies together produce the world [65]. For instance, as a child (participant) receives a ball (object) in their arms, it is not clear where the child begins and the ball ends; or as a child rolls down a gentle slope, the body and the slope stretch out into each other; or as a child wraps a ribbon around their wrist to participate in the Indigenous “Butterfly Dance,” as one of NWOW’s PL-enriched movement resources, the child enacts a touching in togetherness with the ribbon, imaginaries of the butterfly, and with the land on which they dance. New materialism, therefore, is not concerned with the isolated and inert structuring of the human experience (e.g., data that show how PL is experienced by preschool-aged children in the ECEC) but rather, what the data show of relationships. In other words, the focus is not about what matter is but rather, with what matter does through bodily performances with the world as the body is imbued with the affected/affecting relationship [66,67].

While the conversational interviews in NWOW’s pilot project were designed to generate stories from three early childhood educators in NWOW’s pilot project pertaining to ‘knowledge and encounters with PL’, ‘cultural practices rooted in PL’, and ‘enablers and barriers to culturally rooted PL’, in this article, we are not focused on the *representational content* of the stories. Rather, as we move away from representations of data through our methodological positioning of new materialism, we focus on how the stories ignited our researcher bodies with curiosity and inspiration in varying degrees through the intensities of affect. The educator stories included below had their own way of making themselves known to us as they resonated through transformative moments of rupture. We appreciate that we could have included a multitude of other educator stories that were generated from the conversational interviews; yet in this instance, we followed the ‘data that glowed’ [38]. Moreover, as previously discussed, because we are not passive bystanders looking in on our research, we also entangle our researcher stories into what counts as data.

## 4. Moment of Rupture One—A Break in Political and Cultural Discourses

*The sound of our language, whether I can understand every word, is calming and reassuring as it drives me back to the place where I was surrounded by grandmothers and grandfathers, aunts and uncles, and cousins who all shred the memories of our common participation in our Indigenous life and ways. This presence is a continuous source of guidance and support as we lived on Mother Earth and enjoyed the trees, grass, and bushes as our playground within hearing distance of the medicine lodge our Elders relaxed and worked in. These are rare places where nature sets aside a small space for a table to work at or benches to rest on, and the boundary between inside and outside is blurred because there is little need of doors.*

*With the presence of so many Indigenous students, educators, and Elders in the building, this ECEC is one place in the busy city that holds the potential to be one of these rare places that embody the connection of Indigenous peoples to the land. Bright open windows let in the sunlight, or the gray colors of a rainy day, or the pure whiteness of a snowy day. A busy street surrounding the building cannot hold back a parade of Fall leaves as they blow past the windows; and always in the background are all the Indigenous voices of invisible generations and the Indigenous presence of visitors bringing the lifeways into the space.*

*Walls cannot hold out the laughter as Indigenous people support their learning always with laughter. Artwork further brings the distant flora of the outside world into the space. The unique understanding of Indigenous people in their beliefs connect them to their spirit and to the land through the creation is further reflected in artwork on the surfaces; but most important is hearing the language spoken by the Elders in word or song. Words that remind us of who we truly are and songs with the ever-present strong beat of our hearts that push our bodies to move and to love our lives*.

(Wahpepah, Fieldnotes, December 2022)

Many Indigenous and non-Indigenous scholars alike have pointed to the pervasive and ongoing structures of colonialism imbued within political and cultural discourses of Western education [68,69,70]. Within a movement context, colonialism has significantly contributed to a loss of cultural identity and practices of land-based PA that traditionally demanded more robust physical fitness and skill [71,72,73]. The effects of colonialism on both educational processes and practices and the way Indigenous children move their bodies was emphasized by early childhood educators collaborating in NWOW’s pilot project, Harmony and Tulip. (Pseudonyms are used throughout this article, as per university ethics protocols. Names were chosen by the early childhood educators.) Both educators indicated they occasionally facilitated some cultural-based activities in their daily classroom practices, for example, Indigenous dancing; yet they were not familiar with any ECE resources or educational supports that promoted PL through Indigenous Knowledges or through specific Indigenous games and activities. As Harmony said:

*[Indigenous] culture has been absorbed. It’s gone. We are disconnected from [Indigenous] identity. That’s where residential schools come in; they took it [identity] from their parents so then they couldn’t teach it to their kids and then their kids don’t know it and then the babies don’t know it; nobody knows it. People expect you to do be [Indigenous], but you’re not because you were never exposed [to the culture]. My grandpa was in residential school. I’m sad that my mom knows nothing. She should know more than she does. I grew up in Saskatoon and not on a reserve, so I am an outcast from both worlds. And we shouldn’t feel like that. Only one of the children here are exposed to Cree culture at home. So, I want to expose children to their culture so that they can pass it onto their children and so on, you know—give culture back. ‘Cause everyone should have a sense of their culture regardless of what your culture is, you know? You should be provided that opportunity*.

(transcribed interview notes, 2022)

Decolonizing approaches to Indigenous land-based education seek to uncover how settler colonial projects are maintained and reproduced while simultaneously working to recenter Indigenous cosmologies [55,74]. For us in the NWOW initiative, decolonizing approaches are enacted through the positioning of PL alongside Indigenous cultural connections and traditional teachings of land-based practices through a range of learning resources. For example, one of the NWOW learning resources includes a game called ‘Tatanka’ in which children are provided with opportunities to develop movement competence (e.g., running, dodging), confidence, motivation, social skills (e.g., cooperation), body control, and spatial awareness while also engaging with a traditional Indigenous story about the buffalo (Tatanka). As Tulip emphasized, culture plays an integral role within pursuits of health and wellness for Indigenous community members.

*The [Tatanka] story is their own [children’s] story. So, after hearing the story, [the children] are connected with the game and feel more confident to move because it is their story. They can be challenged because they understand the story. After the storytelling when I tell them how to play the game, they feel more interested and confident to participate. We are doing a ‘learning circle’ to tell the story. I see them relating with the cultural story, and then they better focus on their physical development through the story. The body movements are actually from their roots. The pictures [on the learning resources] show connections to land and culture, like the buffalo and the tipi, for example*.

(Transcribed interview notes, 2022)

As the narratives from Harmony and Tulip show, the NWOW PL-enriched resources facilitated movement opportunities through cultural connections and traditional teachings relating to land. Thus, they offered a break—or moment of rupture—in the natural order, the normalizing educational practices that are reified through political and cultural discourses of colonialism. In turn, this break initiated different patterns of PL as contextualized, emplaced, and situated with land; calling us in to good relations (as opposed to calling out) through the grounded, lived, embodied, and embedded practices of knowing/being/thinking/doing/feeling that cultivates, maintains, and sustains affirmative relationships with all earthly other(s) [75] and to account for our relations when they are not good [36]. 

## 5. Moment of Rupture Two—A Break in Social Discourses

Visiting Harmony, Tulip, and Carmen at the ECEC today, I knew I would not be greeted by a bustling classroom full of eager and enthusiastic children but the same two to three children who were consistently present at the ECEC during our collaboration.

**Figure 1:**
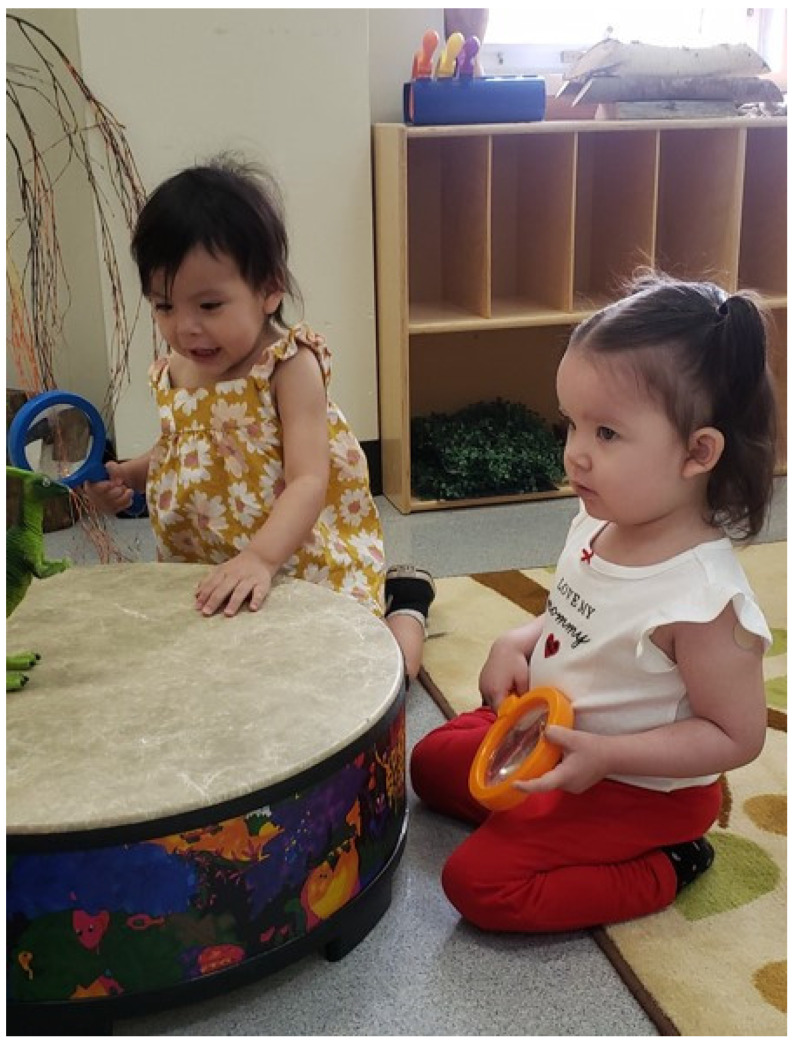
Drumbeats.

*These children’s parents attended the adjacent high school; and while they were still kids themselves, they showed incredible strength and determination to return to high school after facing many disruptions to their education in the face of complex social challenges. As I entered the ECEC, I saw two children playing with the deer-hide drum. Every now and then, they would generate enough momentum to carry out a drumbeat with control and precision before becoming distracted with the ‘lurking’ toy dinosaur (shown in Figure 1). Through informal conversations with teachers at the high school, it became evident that one of the biggest barriers to PA and PL for these children’s parents was the lack of confidence to participate. But this lack of confidence was evident in Harmony and Tulip too; a lack of confidence which meant it was easier to stay indoors and play in more sedentary ways rather than venturing to the playground or the gym to move and thus exposed to the potential of failure or judgement. These children and the children’s parents certainly needed their teachers to actively participate in movement with them; for the teachers to guide and show the children that it was safe to express themselves through movement; that it was safe to fall in love with how their body moved, irrespective of the spectator gaze. Yet what happens when the educators are simply not confident to do so because of their own limiting stories with PA and PL (and perhaps physical education)? Afterall, Harmony did tell me that she wishes she had someone move with her as a child, then she might not be so deterred by movement as an adult*.

(Riley, Fieldnotes, May 2022)

Sedentary lifestyle behaviours are becoming increasingly normalized in our movement-suppressed societies [76,77]. Yet, for early childhood educator, Carmen, it was not just the lack of movement opportunities for preschool-aged children that was of concern but the lack of educational rigor that was provided within any given movement opportunity.

*When the children go outside, they get so excited to move! But they won’t move unless the adults move with them. Sometimes, the adults will tell them to go play and they will inevitably just flock to the sandpit and dig and dig and dig. There is no game. No structure. No bigger purpose. It’s random. I would like them to be able to connect their play and importantly, their movement with deeper learning as prompted by the educators. We need to provide the children with more meaningful movement opportunities. But mostly, we need to be the influence for these children*.

(transcribed interview notes, 2022)

It is important to follow the child’s lead in their expressions of play (E. Stevenson, Personal Communication, 17 November 2022). However, Carmen sought to enhance a more ‘hands on’ and relational emphasis between the educator and the child and possibilities of play to include a more robust focus on active play (without negating the valuable contributions of all types of play for children’s wholistic health and wellness [78,79]) (movement), as role-modeled through early childhood educator efficacy for personal health and wellness in the development of PL [80].

Addressing Carmen’s concerns and prompting a moment of rupture within normalizing practices of the ECEC, NWOW focused on early childhood educator health and wellness through collaborative and relational approaches to active play and movement, as represented in the PL-enriched movement resources. For instance, one of the NWOW resources, ‘Tipi Toss’. facilitated early childhood educator engagement with children’s movement as the educators (and children) are provided with cues and positive challenges for receiving, sending, balance, and coordination; while also being situated within opportunities for embodying Indigenous traditional teachings associated with the tipi as depicted in the ‘Tipi Toss’ story provided by Elder Kathy. Upon learning the meaning behind the tipi poles, the children stand in a circle in their imaginary tipi and toss the ball to each other with the goal of cooperation by looking directly at each person to whom they are throwing the ball. If the targeted child catching the ball drops the ball, they sit in the centre until the next child drops the ball. Once the next child drops the ball, the child in the centre can rejoin the circle. The game continues until only one player remains who has not dropped the ball.

*Living in a small space like a lodge or tipi, especially in cold weather, caused many games to evolve. Balls made of leather scraps were used in a football game that could range far and wide across the plains in warm weather as there were no boundaries. This wouldn’t be possible in snow or stormy weather, but the ball was still a favorite toy. Smaller games with balls can be used inside. Living in small spaces in large family groups, First Nations people taught their children to be respectful and careful of one another’s safety and comfort within the tipi. Tipi teachings were essential and guided family life. Each pole of the tipi represented a teaching within First Nation’s life. The tipi poles represent: obedience, respect, humility, happiness, love, faith, kinship, cleanliness, thankfulness, sharing, strength, good child rearing, hope, and ultimate protection. Inter-connectedness is represented by the tipi flaps, and the rope represents the sacred bond or our connection to the universe.*

Carmen further suggested, “It is not educational policies that drive movement practices in ECECs but the educator’s desire to do so.” (Transcribed interview notes, 2022); however, Tulip indicated that through engagement with NWOW’s resources, she developed a stronger sense of efficacy to engage in active play and movement with the children and with land:

*We know about the tipi; we know about [Indigenous] hunting and dance; we know about the beaver and the deer…but these cards [learning resources] helped us to provide more cultural knowledge in our physical activity. Before, we would just show the children a picture of a beaver or a deer, or of a tipi, and then separately at a later time, we would take the children outside; but now, we are bringing these two things together. We take the children outside to actually be the beaver and the dear and the tipi!*.

(Transcribed interview notes, 2022)

As Tulip’s narratives gather land in PL storying, it is not clear where the child begins and the land ends (relating to the beaver or the dear in this scenario). Thus, bodies are no longer doing movement within the disciplining and limiting constraints of colonial logics; but movement is doing relationships with land [25,31,81]. Barad [32] referred to this process as cutting-together-apart. For Barad, the situated labor of affective materiality initiates a singular and dynamic act of cutting-together-apart as one. As such, affective materiality enacts a living-with/through other(s), which in turn, works to dismantle Cartesian cuts of dichotomy that separates categories and thus, generates a child/PL/land assemblage. The implications of PL emergent within radical relationality is explored next in this final section. 

## 6. (In) Conclusion: The Implications of Physical Literacy Emergent within Radical Relationality

In this case assemblage, we explored some of the contested, complex, and politicized realities of contemporary childhood in confronting some dominant and pervasive political, cultural, and social discourses that impact PL for Indigenous preschool-aged children in NWOW’s pilot project. Through complexity thinking’s radical relationality enabling a more dynamic and contingent way for thinking about social organization without reductionism to unfettered agency or deterministic structures [82], we sought to show how PL can shift from a concept that is defined by social descriptions to a concept that is constituted through social and material relations (e.g., with land). In the context of this case assemblage, the intent was to re-emphasize how PL is a concept to be actualized through relations (with land or otherwise) rather than as something to be represented as separate and discrete from the moving body nor as something that is defined through its essence or nature [83]. That is, while PL is typically understood through four interconnected domains (affective, physical, cognitive, and behavioral), we have deliberately avoided a focus on how the child negotiated these domains in this article to depart from an essentialist narrative that suggests a rigid, fixed, stable, and static definition of PL. In other words, rather than generating knowledge about PL through descriptions of reality that position the human as separate and detached from the world, our understanding of PL is generated from direct material engagement with the world as we prioritized a focus on matters of practices, doings, and actions within performative accounts [66,67].

Disassociating from Euro-centric outcomes-focused ideals and dismantling the stronghold of universalizing, instrumentalist, and predictable developmental trajectories in colonial ideologies, we extend movement pedagogies beyond regulatory governance in this article. We appreciate that regulatory governance is designed to promote more active and healthy children (e.g., the Canadian Physical Activity Guidelines for the Early Years by the Canadian Society for Exercise Physiology [2021] and ParticipACTION Early Years Guidelines [2016]) and equally acknowledge the importance of preschool-aged children having opportunities to learn how to throw and catch, to balance, to jump, skip, gallop, run, and hop in safe and age-appropriate ways. However, grounding PL in co-created, negotiated, complex, and messy experimentations with movement and play as contingent on the situated relationships, means PL is not something to be obtained or achieved sometime out in the future when the learning conditions are right for particular learners; but rather, PL is imminent through radical relationality as the individual comes into being through moment-to-moment enactments of moving with the world. 

As NWOW expands to other collaborations with ECECs across Saskatchewan, an important provocation that has emerged from this pilot project prompts the question of how we might enact politicized, ethical, and critical engagement with and for complex worlds that are imbued with messy and radical asymmetrical power relations. That is, paying attention to the ongoing power imbalances imbued within colonial structures and systems, we acknowledge the scope within (early childhood) education contexts to further grapple with the political nature of education through continued efforts to disrupt universalizing, one-size-fits-all approaches to learning and critical engagement with multiple and diverse worldviews. This provocation (re)affirms the role of appropriate, relevant, and meaningful policy, practice, and research to attend to the particularity and specificity of relationships [84,85]. While the work of this case assemblage gathers land within co-created conditions of PL to promote (Indigenous) preschool-aged children’s wholistic health and wellness, we do not suggest ideas in this article can be easily transferred to inform another context. Differentiating between universalizing and generalizing, however, our hope is that the NWOW’s pilot project might prompt different stories, worldings, and assemblages from different emplaced and situated contexts.

## References

[1] Belanger K., Barnes J.D., Longmuir P.E., Anderson K.D., Bruner B., Copeland J.L., Gregg M.J., Hall N., Kolen A.M., Lane K.N. (2018). The relationship between physical literacy scores and adherence to Canadian physical activity and sedentary behaviour guidelines. BMC Public Health.

[2] Bremer E., Graham J.D., Bedard C., Rodriguez C., Kriellaars D., Cairney J. (2020). The Association Between PLAYfun and Physical Activity: A Convergent Validation Study. Res. Q. Exerc. Sport.

[3] Dudley D., Cairney J., Wainwright N., Kriellaars D., Mitchell D. (2017). Critical Considerations for Physical Literacy Policy in Public Health, Recreation, Sport, and Education Agencies. Quest.

[4] Smith S., O’Grady L., Cubillo C., Cavanagh S. (2017). Using Culturally Appropriate Approaches to the Development of KidsMatter Resources to Support the Social and Emotional Wellbeing of Aboriginal Children. Aust. Psychol..

[5] Wilson S. (2008). Research Is Ceremony: Indigenous Research Methods.

[6] (2016). International Physical Literacy Association. https://www.physical-literacy.org.uk/.

[7] Durden-Myers E.J., Whitehead M.E., Pot N. (2018). Physical literacy and human flourishing. J. Teach. Phys. Educ..

[8] Durden-Myers E.J., Meloche E.S., Dhillon K.K. (2020). The Embodied Nature of Physical Literacy: Interconnectedness of Lived Experience and Meaning. J. Phys. Educ. Recreat. Dance.

[9] Durden-Myers E.J., Bartle G., Whitehead M.E., Dhillon K.K. (2021). Physical Literacy and Intentionality: Embodied Beckoning. J. Phys. Educ. Recreat. Dance.

[10] Whitehead M.E. (2001). The concept of physical literacy. Eur. J. Phys. Educ..

[11] Whitehead M.E. (2010). Physical Literacy: Throughout the Lifecourse.

[12] Whitehead M. (2019). Physical Literacy across the World.

[13] Cairney J., Dudley D., Kwan M., Bulten R., Kriellaars D. (2019). Physical Literacy, Physical Activity and Health: Toward an Evidence-Informed Conceptual Model. Sports Med..

[14] Caldwell H.A., Di Cristofaro N.A., Cairney J., Bray S.R., Macdonald M.J., Timmons B.W. (2020). Physical Literacy, Physical Activity, and Health Indicators in School-Age Children. Int. J. Environ. Res. Public Health.

[15] Azzarito L. (2009). The Panopticon of physical education: Pretty, active and ideally white. Phys. Educ. Sport Pedagog..

[16] Azzarito L. (2010). Future Girls, transcendent femininities and new pedagogies: Toward girls’ hybrid bodies?. Sport Educ. Soc..

[17] Azzarito L. (2012). The rise of the corporate curriculum: Fatness, fitness, and whiteness. Biopolitics and the ’Obesity Epidemic’.

[18] Azzarito L., Macdonald D., Dagkas S., Fisette J. (2017). Revitalizing the Physical Education Social-Justice Agenda in the Global Era: Where Do We Go From Here?. Quest.

[19] Hill J., Azzarito L. (2012). Representing valued bodies in PE: A visual inquiry with British Asian girls. Phys. Educ. Sport Pedagog..

[20] MacNeill M., Rail G. (2010). The visions, voices and moves of young ‘Canadians’: Exploring diversity, subjectivity and cultural constructions of fitness and health. Young People, Physical Activity and the Everyday.

[21] Sanderud J.R., Gurholt K.P., Moe V.F. (2020). ‘Winter children’: An ethnographically inspired study of children being-and-becoming well-versed in snow and ice. Sport Educ. Soc..

[22] Sperka L., Enright E. (2019). And if you can’t hear us?: Students as customers of neo-HPE. Sport Educ. Soc..

[23] Blaise M. (2014). Interfering with Gendered Development: A Timely Intervention. Int. J. Early Child..

[24] Pacini-Ketchabaw V., Taylor A. (2015). Unsettling the Colonial Places and Spaces of Early Childhood Education.

[25] Rotas N., Land N. (2020). Taking up the Practice of Study: A Critical Move in the Break of Early Childhood Movement Pedagogies. Equity Excel. Educ..

[26] Crinall S. (2019). Sustaining Childhood Natures: The Art of Becoming with Water.

[27] Cutter-Mackenzie-Knowles A., Malone K., Barratt Hacking E. (2020). Research Handbook on Childhoodnature: Assemblages of Childhood and Nature Research.

[28] Malone K., Tesar M., Arndt S. (2020). Theorising Posthuman Childhood Studies.

[29] Riley K. (2020). Posthumanist and Postcolonial Possibilities for Outdoor Experiential Education. J. Exp. Educ..

[30] Ovens A., Hopper T., Butler J. (2012). Complexity Thinking in Physical Education.

[31] Riley K., Proctor L. (2022). The senses/sensing relationship in physical literacy: Generating a worldly (re)enchantment for physical education. Sport Educ. Soc..

[32] Barad K. (2007). Meeting the Universe Halfway: Quantum Physics and the Entanglement of Matter and Meaning.

[33] Dolphijn R., van der Tuin I. (2012). New Materialism: Interviews & Cartographies.

[34] Neto L.S., Venâncio L., Ovens A.P. (2021). Special issue—Editorial introduction—Physical education teacher education and its complexities: Mapping contexts for research and sharing experiences from Brazil. Sport Educ. Soc..

[35] Alberga A.S., Fortier M., Bean C., Freedhoff Y. (2019). Youth get a D+ grade in physical activity: How can we change public health messages to help reverse this trend?. Appl. Physiol. Nutr. Metab..

[36] Liboiron M. (2021). Pollution Is Colonialism.

[37] Lather P., St Pierre E.A. (2013). Post-qualitative research. Int. J. Qual. Stud. Educ..

[38] Maclure M. (2013). Researching without representation? Language and materiality in post-qualitative methodology. Int. J. Qual. Stud. Educ..

[39] St Pierre E.A., Jackson A.Y., Mazzei L.A. (2016). New empiricisms and new materialisms: Conditions for new inquiry. Cult. Stud. Crit. Methodol..

[40] Higgins M. (2017). Post-qualitative mo(ve)ments: Concluding remarks on methodological response-abilities and being wounded by thought. Reconceptualizing Educ. Res. Methodol..

[41] Fox N.J., Alldred P. (2017). Sociology and the New Materialism: Theory, Research, Action.

[42] Hughes C., Lury C. (2013). Re-turning feminist methodologies: From a social to an ecological epistemology. Gend. Educ..

[43] De Line S. (2016). All My/Our Relations: Can Posthumanism Be Decolonized? Open! Platform for Art, Culture and the Public Domain. http://onlineopen.org/all-my-our-relations.

[44] Martin B. (2017). Methodology is content: Indigenous approaches to research and knowledge. Educ. Philos. Theory.

[45] Todd Z. (2016). An indigenous feminist’s take on the ontological turn: ‘Ontology’ is just another word for colonialism. J. Hist. Sociol..

[46] Tuck E. A turn to where we already were? Settler inquiry, indigenous philosophy, and the ontological turn. Presented at the Annual Meeting of the American Educational Research Association.

[47] Watts V. (2013). Indigenous place-thought and agency amongst humans and nonhumans (First Woman and Sky Woman go on a European world tour!). Decolonization Indig. Educ. Soc..

[48] Andersson Å., Korp P., Reinertsen A.B. (2020). Thinking With New Materialism in Qualitative Case Studies. Int. J. Qual. Methods.

[49] Bennett J. (2010). Vibrant Matter: A Political Ecology of Things.

[50] Gough N., Adsit-Morris C. (2020). Words (*are*) matter: Generating material-semiotic lines of flight in environmental education research assemblages (with a little help from SF). Environ. Educ. Res..

[51] Roy K. (2003). Teachers in Nomadic Spaces: Deleuze and Curriculum.

[52] Ahenakew C. (2016). Grafting Indigenous ways of knowing onto non-Indigenous ways of being: The (underestimated) challenges of a decolonial imagination. Int. Rev. Qual. Res..

[53] Bang M., Curley L., Kessel A., Marin A., Suzukovich E.S., Strack G. (2014). Muskrat theories, tobacco in the streets, and living Chicago as Indigenous land. Environ. Educ. Res..

[54] Bang M. (2020). Learning on the Move Toward Just, Sustainable, and Culturally Thriving Futures. Cogn. Instr..

[55] Calderon D. (2014). Speaking back to Manifest Destinies: A land education-based approach to critical curriculum inquiry. Environ. Educ. Res..

[56] Wildcat M., McDonald M., Irlbacher-Fox S., Coulthard G. (2014). Learning from the land: Indigenous land based pedagogy and decolonization. Decolonization Indig. Educ. Soc..

[57] Ward S., Chow A.F., Humbert M.L., Bélanger M., Muhajarine N., Vatanparast H., Leis A. (2018). Promoting physical activity, healthy eating and gross motor skills development among preschoolers attending childcare centers: Process evaluation of the Healthy Start-Départ Santé intervention using the RE-AIM framework. Eval. Program Plan..

[58] Chow A.F., Leis A., Humbert L., Muhajarine N., Engler-Stringer R. (2016). Healthy Start—Départ Santé: A pilot study of a multilevel intervention to increase physical activity, fundamental movement skills and healthy eating in rural childcare centres. Can. J. Public Health.

[59] Riley K., Froehlich Chow A., Humbert M.L., Houser N., Brussoni M., Erlandson M. Etuaptmumk (Two-eyed Seeing) in Nature’s Way-Our Way: Braiding physical literacy and risky/adventurous play through Indigenous games, activities, cultural connections, and traditional teachings. AlterNative in press.

[60] Macdonald C., Steenbeek A. (2015). The Impact of Colonization and Western Assimilation on Health and Wellbeing of Canadian Aboriginal People. Int. J. Reg. Local Hist..

[61] McGuire-Adams T. (2021). “This is what I heard at Naicatchewenin”: Disrupting embodied settler colonialism. J. Indig. Wellbeing.

[62] Drahota A., Meza R.D., Brikho B., Naaf M., Estabillo J.A., Gomez E.D., Vejnoska S.F., Dufek S., Stahmer A.C., Aarons G.A. (2016). Community-Academic Partnerships: A Systematic Review of the State of the Literature and Recommendations for Future Research. Milbank Q..

[63] Hergenrather K.C., Geishecker S., McGuire-Kuletz M., Gitlin D.J., Rhodes S.D. (2010). An Introduction to Community-Based Participatory Research. Rehabil. Educ..

[64] Seigworth G.J., Gregg M., Gregg M., Seigworth G.J. (2010). An inventory of shimmers. The Affect Theory Reader.

[65] Coole D., Frost S. (2010). New Materialisms.

[66] Fullagar S. (2017). Post-qualitative inquiry and the new materialist turn: Implications for sport, health and physical culture research. Qual. Res. Sport Exerc. Health.

[67] Markula P. (2019). What is New About New Materialism for Sport Sociology? Reflections on Body, Movement, and Culture. Sociol. Sport J..

[68] Nxumalo F. (2016). Towards ‘refiguring presences’ as an anti-colonial orientation to research in early childhood studies. Int. J. Qual. Stud. Educ..

[69] Tuck E., Gaztambide-Fernandez R.A. (2013). Curriculum, replacement, and settler futurity. J. Curric. Theor..

[70] Tuck E., McKenzie M. (2015). Place in Research: Theory, Methodology and Methods.

[71] Forsyth J. (2007). The Indian Act and the (re) shaping of Canadian Aboriginal sport practices. Int. J. Can. Stud./Rev. Int. D’études Can..

[72] Paul S., Jones G., Jakobi J. (2019). Deer hunting: An innovative teaching paradigm to educate Indigenous youth about physical literacy. J. Indig. Wellbeing.

[73] McGuire-Adams T.D., Adams R.J. (2015). 4Vitality kettle bell training: Fostering physical resurgence amongst the urban Indigenous community members. Rev. Phéneps/PHEnex J..

[74] Battiste M. (2013). Decolonizing Education: Nourishing the Learning Spirit.

[75] TallBear K. (2014). Standing with and speaking as faith: A feminist-indigenous approach to inquiry. J. Res. Pract..

[76] Hidding L.M., Altenburg T.M., Chinapaw M.J.M., Van Ekris E. (2017). Why Do Children Engage in Sedentary Behavior? Child- and Parent-Perceived Determinants. Int. J. Environ. Res. Public Health.

[77] Owen N., Sugiyama T., Eakin E.E., Gardiner P.A., Tremblay M.S., Sallis J.F. (2011). Adults’ Sedentary Behavior: Determinants and Interventions. Am. J. Prev. Med..

[78] Herrington S., Brussoni M. (2015). Beyond Physical Activity: The Importance of Play and Nature-Based Play Spaces for Children’s Health and Development. Curr. Obes. Rep..

[79] Ramsden R., Han C.S., Mount D., Loebach J., Cox A., Herrington S., Bundy A., Fyfe-Johnson A., Sandseter E.B.H., Stone M. (2022). An Intervention to Increase Outdoor Play in Early Childhood Education Centers (PROmoting Early Childhood Outside): Protocol for a Pilot Wait-list Control Cluster Randomized Trial. JMIR Res. Protoc..

[80] Lugossy A.-M., Chow A.F., Humbert M.L. (2022). Learn to Do by Doing and Observing: Exploring Early Childhood Educators′ Personal Behaviours as a Mechanism for Developing Physical Literacy Among Preschool Aged Children. Early Child. Educ. J..

[81] Land N., Danis I. (2016). Movement/ing Provocations in Early Childhood Education. J. Child. Stud..

[82] Ovens A. (2017). Putting Complexity to Work to Think Differently about Transformative Pedagogies in Teacher Education. Issues Teach. Educ..

[83] Ovens A., Enright E. (2021). Infection or affection: Physical literacy and the reterritorialisation of the HPE curriculum. Curric. Stud. Health Phys. Educ..

[84] Coulthard G.S. (2014). Red Skin, White Masks: Rejecting the Colonial Politics of Recognition.

[85] Deloria V. (1999). Spirit & Reason: The Vine Deloria, Jr., Reader.

